# Unexpected histopathology results following routine examination of cholecystectomy specimens: How big and how significant?

**DOI:** 10.1016/j.amsu.2020.11.019

**Published:** 2020-11-13

**Authors:** Abdulkarim Hasan, Khalid Nafie, Mohammed Yousef Aldossary, Amal Ismail, Khaled Monazea, Mohamad Baheeg, Kamal Rady, Reda Elhawary, Adel A. Ibrahim

**Affiliations:** a Department of Pathology, Faculty of Medicine, Al-Azhar University, Cairo, Egypt; b Laboratory & blood bank Department, Prince Mishari bin Saud Hospital, Baljurashi, Saudi Arabia; c Department of General Surgery, King Fahad Specialist Hospital-Dammam, Dammam, Saudi Arabia; d Department of Pharmacy Practice, Unaizah College of Pharmacy, Qassim University, Saudi Arabia; e Department of Surgery, Faculty of Medicine, Al-Azhar University, Assiut branch, Egypt; f Department of Surgery, Faculty of Medicine, Al-Azhar University, Cairo, Egypt; g Department of Anatomy and Embryology, Faculty of Medicine, Al-Azhar University, Cairo, Egypt; h Department of Surgical Oncology, Faculty of Medicine, Al-Azhar University, Cairo, Egypt

**Keywords:** Cholecystectomy, Cholecystitis, Gallbladder carcinoma, Histopathological examination

## Abstract

**Background:**

Routine histopathological examination (RHPE) of all gallbladder specimens is required to detect the presence of gallbladder carcinoma (GBC) or any other pathology. The work aims to study the incidence and the clinical significance of detecting unusual gallbladder findings upon the RHPE of the referred cholecystectomy specimens to a histopathology laboratory section at a referral hospital in Saudi Arabia during one year period.

**Materials and methods:**

From May 2019 to May 2020, all histopathology reports of 444 consecutive gallbladder specimens after elective and emergency cholecystectomies were retrospectively analyzed and divided into two groups; usual findings and unusual findings which were reviewed blindly by two other pathology consultants. Frequencies, descriptive statistics, normality test, and correlations were run. The Interrater reliability between clinical and histopathological diagnosis was assessed statistically by kappa test.

**Results:**

The results of histopathological examination of these gallbladder specimens showed that chronic cholecystitis was found in 296 out of 444 total cases (66.7%), acute cholecystitis in 52 cases (11.7%), and other associated usual findings in 85 cases (19%). Three cases (0.7%) of incidental carcinomas and other three cases (0.7%) of dysplasia. Eosinophilic carcinomas were detected in two cases (0.45%), gallbladder complete septum was found in one case, and one case of Phrygian cap anomaly. All patients with gallbladder carcinoma were diagnosed incidentally during the histopathological examination.

**Conclusions:**

RHPE of cholecystectomy materials are required to confirm the final diagnosis and document any other pathology. Failure to detect incidental occult carcinoma may be catastrophic, given the poor prognosis.

## Highlights

• Unusual histopathology results were seen in 2.3% of the routinely examined cholecystitis cases.
• Gallbladder carcinoma and also some rare lesions can be accidentally discovered during pathology examination.
• Routine histopathological examination of cholecystectomies in our hospital during one year showed a significant clinical value.

## 1 Introduction

Cholecystectomy for clinical diagnosis of cholecystitis is one of the common surgical procedures worldwide [1]. RHPE of cholecystectomy materials are required to confirm the final diagnosis and document any other pathology, so all gallbladder specimens surgically removed during cholecystectomy are traditionally sent for histopathologic examination [2,3]. Several recent studies questioned the necessity for RHPE of all gallbladder specimens reporting that selective histopathology examination is feasible and safe [4]. Although the yield of RHPE of gallbladder specimens is relatively low, the consequences of histopathological alteration such as intraepithelial neoplasia or gallbladder carcinoma are immense. Failure to detect incidental occult carcinoma may be catastrophic, given the poor prognosis [5]. Despite the incidence of GBC in Asian countries (8.1 per 100 000) is much higher than the Western world (0.7 per 100 000), there are some tertiary hospitals in Asia agreed with the practice of discarding the gallbladder specimen when appears macroscopically unremarkable, this practice depends on the assumption that GBC is always associated with naked-eye abnormalities. However, the incidental GBC (without any macroscopic suspicion) is not rare [6,7]. The incidental carcinoma is not the only unexpected histopathology result with clinical importance. Eosinophilic cholecystitis is also an accidentally found diagnosis during RHPE, which may be associated with eosinophilic gastroenteritis or other systemic diseases [8,9]. The usual cholecystitis could be initiated by a congenital anomaly like gallbladder septa, which may not be easily detected during the clinical or the radiological examination. The association with bile duct congenital anomalies should be considered, so that further investigations might be essential [10]. Therefore, this work aimed to study the incidence and significance of detecting usual and unusual gallbladder findings upon the RHPE of the referred cholecystectomy specimens to a histopathology laboratory section at a referral hospital during one year from May 2019 to May 2020.

## 2 Material and methods

This is a retrospective cohort study performed at Prince Mishari bin Saud Hospital in Saudi Arabia , in which the policy of RHPE of all removed gallbladder specimens is followed. Ethical approval was obtained from the Institutional Review Board. Inclusion criteria were all cholecystectomy specimens sent to the histopathology department for the clinical picture of cholecystitis or cholelithiasis from May 2019 to May 2020 that were histopathologically examined in our hospital. Patients with any radiological or clinical (preoperative or intraoperative) suspicion of lesion other than the usual cholecystitis or gallbladder stone disease were excluded. Histopathology request form, containing demographic and clinical data, usually accompanies each received specimen in the laboratory [11]. The request forms, histology reports and medical files of the studied cases were analyzed for recording patient age, sex, clinical diagnosis, surgical procedure, gallbladder length in gross pathology examination, gallbladder diameter, wall thickness, and the final diagnosis. The final diagnoses were divided into two groups; 1) Usual findings including chronic cholecystitis, cholelithiasis, chronic active cholecystitis, and acute cholecystitis in addition to xanthomatous reaction, gangrenous gallbladder, cholesterolosis or any type of metaplasia. 2) Unusual findings, including congenital anomalies, eosinophilic cholecystitis, dysplasia, and malignancy. The eosinophilic cholecystitis and the malignant cases (incidental carcinoma) slides were reviewed blindly by two other pathology consultants, and they agreed with the issued diagnosis of all the revised cases. We report the results of this analysis in accordance with the STROCCS reporting statements [12].

### 2.1 Statistical analysis

Data were transferred into an excel sheet (Excel, Microsoft Corporation, Redmond, WA, USA) and coded. All statistical analyses were performed using the Statistical Package for the Social Sciences (SPSS) version 25 software (SPSS Inc., Chicago, IL, USA). Frequencies, descriptive statistics, normality test, and correlations were run. The Interrater reliability between clinical and histopathological diagnosis was assessed statistically by kappa test.

## 3 Results

This study includes 444 gallbladder histopathological cases that presented to our hospital laboratory during a period of one year from May 2019 to May 2020. The age of involved cases ranged from 18 to 87 years old, with 34.7 median and 15.8 Standard deviation (Table 1). Of cases 330 (74.3%) were females and 114 (25.7%) were males. Clinical data of the reports showed that 360 (81.1%) of the cases were diagnosed as chronic cholecystitis or cholelithiasis, while 84 (18.9%) acute cholecystitis.

**Table 1:** Descriptive statistics of cases according to histopathological finding.

Histopathology diagnosis findings		Range	Minimum	Maximum	Mean	Std. Deviation
Usual	Age	69	18	87	34.65	15.856
	Length	9	5.0	14	7.698	1.78
	Diameter (cm)	4.5	1.5	6.0	2.718	0.6391
	Wall thickness (cm)	1.8	0.2	2	0.4175	0.26435
Unusual	Age	47	22	69	44.44	14.842
	Length	4	7.0	11.0	8.389	1.5366
	Diameter	1.5	2.5	4.0	3.000	0.4330
	Wall thickness	0.9	0.3	1.2	0.489	0.29768

**Fig. 1:**
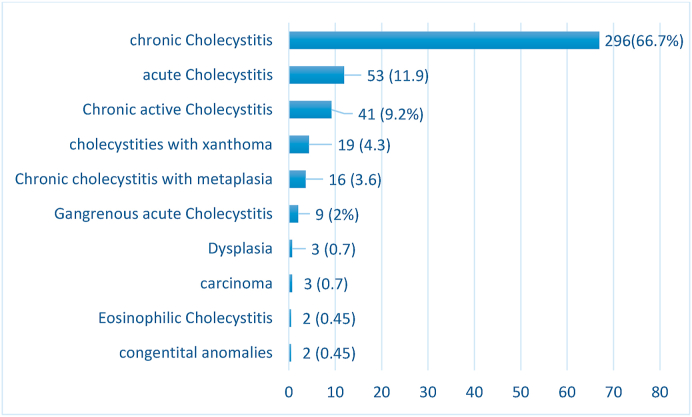
Histopathological diagnosis issued for the total 444 studied cases.

Laparoscopic cholecystectomy was the mode of surgical removal in 406 (91.5%) cases while 26 (5.8%) required open surgery for removal of the gallbladder and 12 (2.7%) cases were transformed from laparoscopic to open during the surgery. Upon histopathological examination 10 (2.3%) of cases showed unusual findings compared to 434 (97.7%) usual findings (Fig. 1).

**Fig. 2:**
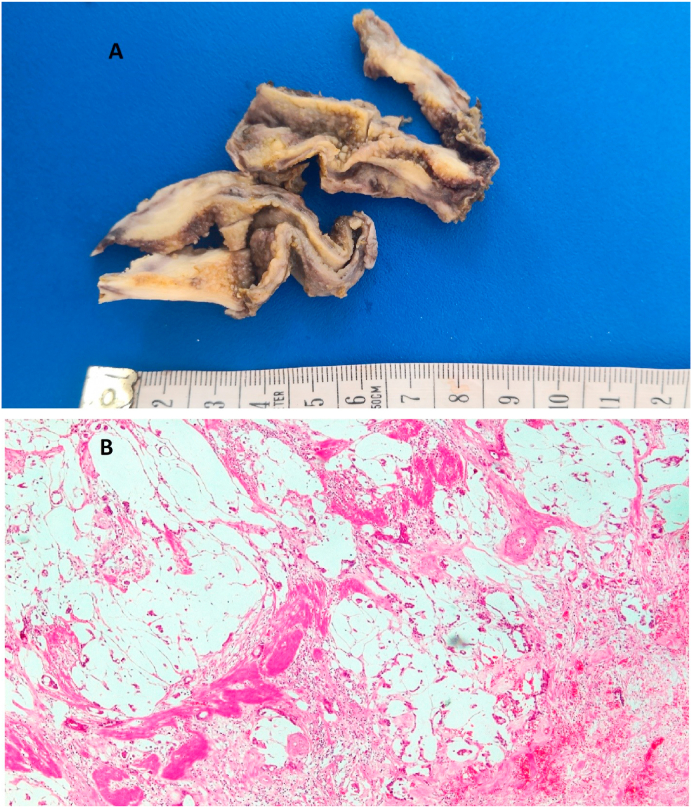
Gall bladder mucinous carcinoma (A) Gross image: Thickened gallbladder wall with glistening white cut section (B) Microscopic image: invasive mucinous carcinoma formed of extracellular mucin pools entangling clusters of tumor cells (H&E stain, X 40).

**Fig. 3:**
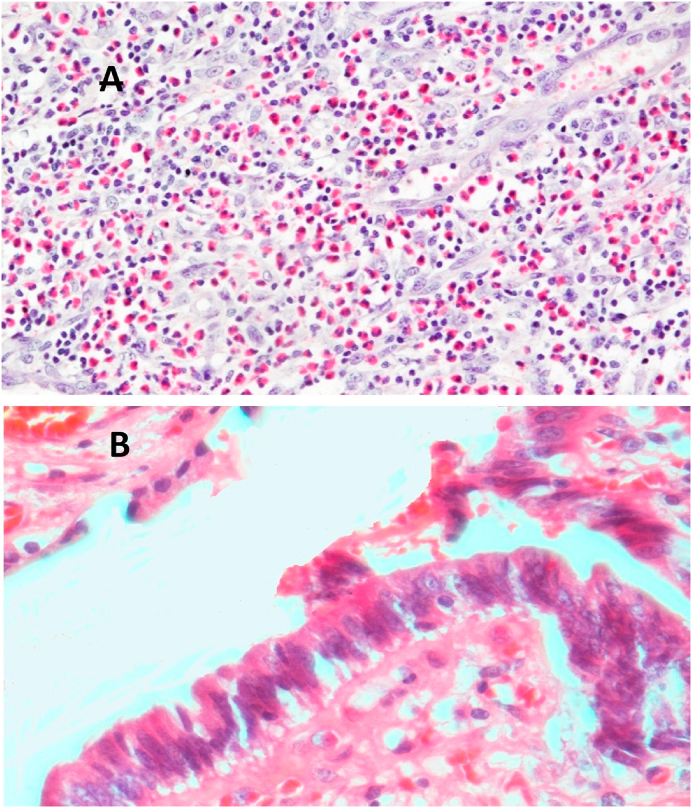
A) A histopathology picture of eosinophilic cholecystitis case showing gall bladder wall mainly infiltrated by eosinophils (H&E stain, X 100). B) A histopathology picture of gall bladder dysplasia mucosa showing dysplasia (H&E stain, X 400).

**Fig. 4:**
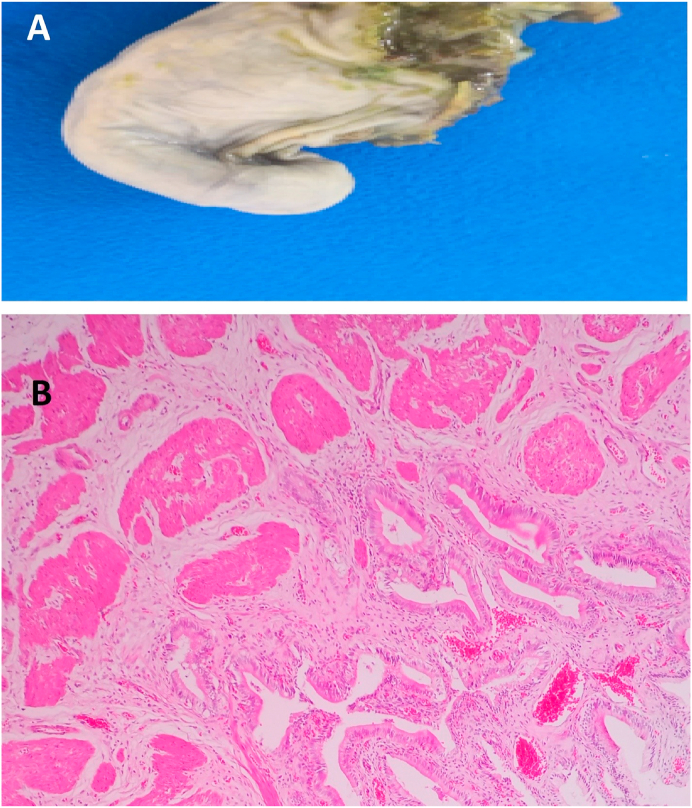
A) A gross picture of a case of Phrygian cap. B) The histopathology picture of the Phrygian cap case showing chronic cholecystitis with mildly dilates blood vessels and scattered inflammatory cells (H&E stain, X 40).

Histological diagnosis of the cases showed 296 (66.7%) chronic cholecystitis versus 53 (11.9%) acute cholecystitis, 85 (19%) other associated usual findings, Three cases (0.7%) of incidental carcinomas (Fig. 2), three cases (0.7%) of dysplasia, two cases (0.45%) of eosinophilic cholecystitis (Fig. 3), one case of gallbladder complete septum, and one case of Phrygian cap anomaly (Fig. 4).

The clinical–histopathological agreement percentage for detection of the usual and unusual findings was 87.3%. The applied Interrater reliability Cohen's kappa coefficient (κ) showed moderate agreement p (0.000) (Table 2). It was suggested by Cohen that Kappa result be interpreted as follows: values ≤ 0 as indicating no agreement and 0.01–0.20 as none to slight, 0.21–0.40 as fair, 0.41–0.60 as moderate, 0.61–0.80 as substantial, and 0.81–1.00 as almost perfect agreement [13]. The p-value considered significant for less than (0.05).

**Table 2:** Interrater reliability test by Cohen's kappa.

Number (444)		Value	Asymp. Std. Error^a^	Approx. T^b^	Approx. Sig.
Measure of Agreement	Kappa	0.482	0.036	16.188	.000

a Not assuming the null hypothesis.

b Using the asymptotic standard error assuming the null hypothesis.

Out of total unusual cases (66.7%) showed more than 8 cm length and more than 0.3 cm in thickness. By applying normality test it was found that data are not following a normal distribution. The non-parametric spearman's test was displayed for correlations and showed that neither involved cases age, sex, nor gallbladder length, thickness, and diameter were statistically significant correlating with unusual findings in histological diagnosis p-values (0 .9, 0.6, 0.112, 0.3, 0.154) respectively.

## 4 Discussion

Gallbladder disorders are the most common diseases in general surgery [14]. The most common type of these disorders is cholecystitis [15], mostly caused by cholelithiasis, which means the development of gallstones [16]. The risk factors of gallbladder diseases include age, female gender, metabolic syndrome, and rapid weight loss [17]. The mean age of the cholecystitis patients in this study is 34.7 ± 15.8 years ranging from 17 to 87 years, slightly higher than that reported by Siddiqui et al., in 2013 [7], Ali et al., in 2010 [18], and lower than what was reported by Olthof in 2018 [19]. Thirty-fourths of the studied cases were female. This female predominance was recorded by several studies [5], some recorded seven eighties female cases, one of them performed in Saudi Arabia [7,20]. To our knowledge, no studies reported male predominance in cholecystectomy operations. Chronic cholecystitis was the leading cause of the surgical operation in 81% of the cases, while the rest 19% were complaining of acute cholecystitis pictures at the time of operation. Acute cholecystitis is the most common complication of cholelithiasis, accounting for 14%–30% of cholecystectomy operations performed in many countries [21,22]. Cholelithiasis may mandate cholecystectomy, especially in symptomatic patients or patients having complications of the gallstones [23]. Cholecystectomy is usually laparoscopically performed, adopting the conventional four-ports technique or using a single or two ports that are modifications of the conventional procedure [24]. Laparoscopic cholecystectomy procedure has some complications such as bowel injury, bile duct injury, vascular injury, adhesions, and port site complications [25]. More than 90% of the studied gallbladders were surgically removed laparoscopically with no recorded complications during the operation, 2.7% of cases were complicated due to acute phase of the cholecystitis or due to adhesions that leaded to conversion to open cholecystectomy, while the rest 5.8% of the cases were surgically opened from the start. Siddiqui et al., in 2013 studied 220 patients with cholecystectomy for gallstones; all of them were performed laparoscopically with few cases transformed into an open operation [7]. The Royal College of Pathologists recommends sending all the removed gallbladder specimens for RHPE due to significant pathology, which could be present even in a normal-appearing specimen [26]. But a few recent studies reported that the yield of RHPE of all removed gallbladder specimens is relatively low in spite of the consequences of histopathological alteration such as GBC and biliary intraepithelial neoplasia are immense [5,19,27]. GBC remains a rare gastrointestinal cancer with an extremely poor prognosis. The high aggressive biological nature of this carcinoma, absence of reliable biomarkers, lack of screening tools, in addition to the late onset of symptoms even in an advanced stage, result in this poor prognosis of the GBC [[28], [29], [30], [31], [32], [33]]. Early detection and studying of the pathology of primary and metastatic carcinomas are challenging [34]; in case of GBC radiologists should examine the gallbladder of patients who are at a greater risk of cancer developing in its entirety trying to find out any abnormality [35].

The incidence rate of GBC in western countries is low (0.7 per 100 000) compared to the high rates in Asia (8.1 per 100 000) [6,[31], [32], [33]]. This geographic differences in tumor incidence are likely related to differences in genetic predisposition, environmental exposures to specific chemicals and regional intrinsic risk of arcinogenic factors that predispose to the GBC [36].

The national data on GBC in the Kingdom of Saudi Arabia has not been analyzed, but it is not among the top 10 cancers in the kingdom [31]. Like various risky tumors, GBC is mainly asymptomatic until it metastasizes or reaches an advanced phase, and if the patients are symptomatic, symptoms are often non-specific [[31], [32], [33],[37], [38], [39], [40]]. The aggressive nature of GBC and the difficulty in early diagnosis present the need to understand this tumor and avoid missing it [[31], [32], [33]]. Three GBC cases in this study are discovered accidentally during one year. The eldest patient was 69 years old female with acute cholecystitis clinical picture, gross examination revealed thickened, and indurated gallbladder wall and finally diagnosed as mucinous carcinoma with stage pT2b. The second case was 65 years old, female, diagnosed clinically as case of gallstones disease, histopathological examination showed fundal polypoid mass 2 × 1.8 cm, and the microscopic examination confirmed moderately differentiated adenocarcinoma with stage pT1b. The youngest patient was a female 55 years old with a thickened gallbladder wall with serosal deposits, which was described histologically as an adenocarcinoma with stage pT3. Some previous retrospective studies in other countries reported zero accidentally discovered carcinomas during the RHPE of the cholecystectomies [27,41]. In contrast, Yaylak et al. in Turkey reported 1/429 (0.2%) incidental gallbladder cancer [1] and others reported incidental carcinomas in more than 2% of the routinely examined gallbladder specimens [7]. According to current guidelines, the procedure of cholecystectomy is sufficient for the stage T1aN0 cancer, but radical cholecystectomy with enbloc resection of the adjacent hepatic parenchyma with regional lymphadenectomy is advised for tumors beyond this stage [42]. Unusual gallbladder findings other than GBC could be detected during the RHPE, such as the congenital anomalies, which are numerous ranging from anomalies of the shape of a number, location, and complete absence or shape anomalies [23]. A Phrygian cap is a congenital abnormality in the shape of the gallbladder with an incidence of 4%, usually diagnosed by cholescintigraphy and multi-phase magnetic resonance imaging, as computerized tomography scan, and ultrasonography are not always conclusive [43]. It is a benign anatomical abnormality, commonly asymptomatic unless complicated with calculus cholecystitis; however, it can be misdiagnosed as gallbladder stones on B-mode ultrasonography that might lead to unnecessary surgical intervention [43]. It seems not to be a high clinical significance to detect a Phrygian cap anomaly during a RHPE of gallbladder specimen. Gallbladder septa or multiseptated gallbladder is another congenital anomaly that is rare and likely secondary to incomplete vacuolization of the gallbladder bud [44]. The septa may be incomplete or complete, involving the entire lumen [45]. Clinically, gallbladder septa can be asymptomatic but commonly present with colicky abdominal pain or biliary pain [46]. During RHPE of the cases in this study, one Phrygian cap gallbladder was detected and a complete septum in a gallbladder also seen in one case showing all histological layers of the gallbladder in addition to the inflammatory infiltrate. Follow-up at the same hospital and further investigations of the two patients revealed no other congenital anomalies. Also, two eosinophilic cholecystitis cases were detected in two cholecystectomy specimens sent to our pathology unit with a clinical diagnosis of acute cholecystitis of two female patients 21 and 44 years old, follow-up of the latest case gave a past history of recurrent gastric upsets that led to a further medical examination which suggested eosinophilic gastritis. The etiology of eosinophilic cholecystitis still remains unknown [9]. It is sometimes associated with other more dangerous diseases such as hypereosinophilic syndrome, eosinophilic-myalgia syndrome, parasitic infestations, and certain drugs [[47], [48]]. No clinical significance was detected for age, sex, nor gallbladder length, thickness, and diameter correlating with unusual histopathological findings. The clinical–histopathological agreement percentage in our hospital is high (87.3%) for the detection of the usual and unusual findings. However, clinicians cannot rely on the clinical or radiological diagnosis of gallbladder diseases in proper managing of the patients.

### 4.1 Strengths and limitations

Lower prevalence of GBC in Saudi Arabia and a small sample size often have limited studies on this subject. One of the major limitations of the study is that it is restricted to patients from a single clinical site. Larger, multi-centric studies are required to obtain a clear picture about the outcome of the RHPE approach in detecting these cancer cases.

## 5 Conclusion

There are different approaches about the examination of gallbladder specimens in the literature. The selective approach is usually performed in areas where the prevalence of GBC is low. However, the RHPE is generally performed in areas where the prevalence of GBC is high. In our study, all of the patients were found to have advanced GBC. Simple Laparoscopic cholecystectomy was not sufficient, and additional interventions were required. We believe that RHPE is necessary for all gallbladder specimens to detect these cancer cases, even in the absence of radiological, clinical, and macroscopic suspicious features.

## Ethical approval

Ethical approval was obtained from the Institutional Review Board (IRB) of the King Fahad Specialist Hospital-Dammam, Dammam, Saudi Arabia. The ethical approval was issued an IRB Study Number – SUR0344.

## Funding sources

This study did not receive any funding from public or private sectors.

## Author contribution

Study concept or design: AH, KM, MB.

Data collection: AH, KN, KM, RE.

Data interpretation: AH, KN, AI, MB, KR, RE.

Literature review: AH, MYD, AI, MB, KR.

Data analysis: KN, AI, AAI.

Drafting of the paper: AH, MYD, AI, KM.

Editing of the paper: AH, MYD, KR, AAI.

Manuscript revision: ALL.

## Registration of research studies

Researchregistry 5966.

## Guarantor

Dr. Abdulkarim Hasan.

## Consent

Not applicable.

## Declaration of competing interest

The authors declare no competing interests.

## References

[1] Yaylak F.A., Deger A.Y., Bayhan Z., Kocak C., Zeren S.E., Kocak F.E., Ekici M.F., Algın M.C. (1971). Histopathological gallbladder morphometric measurements in geriatric patients with symptomatic chronic cholecystitis. Ir. J. Med. Sci..

[2] Agarwal A.K., Kalayarasan R., Singh S., Javed A., Sakhuja P. (2012). All cholecystectomy specimens must be sent for histopathology to detect inapparent gallbladder cancer. HPB.

[3] Deng Y.L., Xiong X.Z., Zhou Y., Shrestha A., Li F.Y., Cheng N.S. (2015). Selective histology of cholecystectomy specimens-is it justified?. J. Surg. Res..

[4] Rathanaswamy S., Misra S., Kumar V., Chintamani, Pogal J., Agarwal A., Gupta S. (2012). Incidentally detected gallbladder cancer - the controversies and algorithmic approach to management. Indian J. Surg..

[5] Corten B.J., Leclercq W.K., Roumen R.M., van Zwam P.H., Dejong C.H., Slooter G.D. (2020). Histological examination of the gallbladder following routine cholecystectomy? A selective analysis is justified. Eur. J. Surg. Oncol..

[6] Mehrotra R., Tulsyan S., Hussain S., Mittal B., Saluja S.S., Singh S., anwar P., Khan A., Javle M., Hassan M.M., Pant S. (2018). Genetic landscape of gallbladder cancer: global overview. Mutation Research/Reviews in Mutation.

[7] Siddiqui F.G., Memon A.A., Abro A.H., Sasoli N.A., Ahmad L. (2013). Routine histopathology of gallbladder after elective cholecystectomy for gallstones: waste of resources or a justified act?. BMC Surg..

[8] Kuwahara T., Kobayashi Y., Yun Y., Kanda A., Asako M., Ueki S., Iwai H. (2019). Eosinophilic cholecystitis occurred in a patient with refractory eosinophilic airway inflammation: a case report. Allergy & Rhinology.

[9] Hasan A., Nafie K. (2019). Eosinophilic cholecystitis: a rare cause of acute cholecystitis ‘case report’. Al-Azhar Assiut Medical Journal.

[10] Tan C.E., Howard E.R., Driver M., Murray-Lyon I.M. (1993). Non-communicating multiseptate gall bladder and choledochal cyst: a case report and review of publications. Gut.

[11] Hasan A., Nafie K., Abbadi O. (2020). Histopathology laboratory paperwork as a potential risk of COVID-19 transmission among laboratory personnel. Infection Prevention in Practice.

[12] Agha R., Abdall-Razak A., Crossley E., Dowlut N., Iosifidis C., Mathew G., for the Strocss Group (2019). The STROCSS 2019 guideline: strengthening the reporting of cohort studies in surgery. Int. J. Surg..

[13] McHugh M.L. (2012). Interrater reliability: the kappa statistic. Biochem. Med..

[14] Knab L.M., Boller A.M., Mahvi D.M. (2014). Cholecystitis. Surgical Clinics.

[15] Russo M.W., Wei J.T., Thiny M.T., Gangarosa L.M., Brown A., Ringel Y., Shaheen N.J., Sandler R.S. (2004). D**igestive and liver diseases statistics**. Gastroenterology.

[16] Petersen J.M., Knight T.T. (1995). Gunshot cholecystitis. J. Clin. Gastroenterol..

[17] Alsaif F.A., Alabdullatif F.S., Aldeghaither M.K., Alnaeem K.A., Alzamil A.F., Alabdulkarim N.H., Aldohayan A.D. (2020). Incidence of symptomatic cholelithiasis after laparoscopic sleeve gastrectomy and its association with rapid weight loss. Saudi J. Gastroenterol..

[18] Ali S.A., Tahir S.M., Soomoro A.G., Siddiqui A.J., Memon A.S. (2010). Open cholecystectomy without intraperitoneal drainage. J. Ayub Med. Coll. Abbottabad.

[19] Olthof P.B., Metman M.J., de Krijger R.R., Scheepers J.J., Roos D., Dekker J.W. (2018). Routine pathology and postoperative follow-up are not cost-effective in cholecystectomy for benign gallbladder disease. World J. Surg..

[20] Murshid K.R. (1998). Symptomatic gallstones: a disease of young Saudi women. Saudi J. Gastroenterol..

[21] Gomes C.A., Junior C.S., Di Saveiro S., Sartelli M., Kelly M.D., Gomes C.C., Gomes F.C., Corrêa L.D., Alves C.B., de Fádel Guimarães S. (2017). Acute calculous cholecystitis: review of current best practices. World J. Gastrointest. Surg..

[22] Orlando R., Russell J.C., Lynch J., Mattie A. (1993). Laparoscopic cholecystectomy. A statewide experience. The Connecticut laparoscopic cholecystectomy registry. Arch. Surg..

[23] Mohammed A.A., Arif S.H. (2019). Midline gallbladder makes a challenge for surgeons during laparoscopic cholecystectomy; case series of 6 patients. Annals of Medicine and Surgery.

[24] Poon C.M., Chan K.W., Lee D.W., Chan K.C., Ko C.W., Cheung H.Y., Lee K.W. (2003). Two-port versus four-port laparoscopic cholecystectomy. Surgical Endoscopy and Other Interventional Techniques.

[25] Deziel D.J., Millikan K.W., Economou S.G., Doolas A., Ko S.T., Airan M.C. (1993). C**omplications of laparoscopic cholecystectomy: a national survey of 4,292 hospitals and an analysis of 77,604 cases**. Am. J. Surg..

[26] Royal College of Pathologists (2005). Histopathology and Cytopathology of Limited or No Clinical Value. Report of Working Group of the Royal College of Pathologists.

[27] Benkhadoura M., Elshaikhy A., Eldruki S., Elfaedy O. (2019). Routine histopathological examination of gallbladder specimens after cholecystectomy: is it time to change the current practice?. Turkish journal of surgery.

[28] Furlan A., Ferris J.V., Hosseinzadeh K., Borhani A.A. (2008). Gallbladder carcinoma update: multimodality imaging evaluation, staging, and treatment options. AJR Am. J. Roentgenol..

[29] Miller G., Jarnagin W.R. (2008). Gallbladder carcinoma. Eur. J. Surg. Oncol..

[30] Glauser P.M., Strub D., Käser S.A., Mattiello D., Rieben F., Maurer C.A. (2010). Incidence, management, and outcome of incidental gallbladder carcinoma: analysis of the database of the Swiss association of laparoscopic and thoracoscopic surgery. Surg. Endosc..

[31] Aldossary M.Y., Alayed A.A., Amr S.S., Alqahtani S., Alnahawi M., Alqahtani M.S. (2018). Gallbladder cancer in Eastern Province of Saudi Arabia: a retrospective cohort study. Annals of medicine and surgery.

[32] Aldossary M.Y., AlQattan A.S., Alghamdi Y.M., Alayed A.A., Alquraish F., AlAnzi O.A., Alabdulrahim N., Alateeq A., Alqahtani M.S. (2019). Surgical outcomes of primary carcinosarcoma of the gallbladder after curative resection: a rare case series. International journal of surgery case reports.

[33] Aldossary M.Y., Alayed A.A., Amr S., Alqahtani M.S. (2018). Primary squamous cell carcinoma of the gallbladder: report of a rare neoplasm from the Eastern Province of Saudi Arabia. International journal of surgery case reports.

[34] Hasan A., Youssef A. (2020). Infiltrating duct carcinoma of the breast; histological difference between the primary and the axillary nodal metastasis. Revista de Senología y Patología Mamaria..

[35] Dwivedi A.N.D., Jain S., Dixit R. (2015). Gall bladder carcinoma: aggressive malignancy with protean loco-regional and distant spread. World Journal of Clinical Cases: WJCC.

[36] Lazcano Ponce E.C., Miquel J.F., Muñoz N., Herrero R., Ferrecio C., Wistuba I.I., De Ruiz P.A., Urista G.A., Nervi F. (2001). Epidemiology and molecular pathology of gallbladder cancer. Ca - Cancer J. Clin..

[37] Cavallaro A., Piccolo G., Di Vita M., Zanghì A., Cardì F., Di Mattia P., Barbera G., Borzì L., Panebianco V., Di Carlo I., Cavallaro M. (2014). Managing the incidentally detected gallbladder cancer: algorithms and controversies. Int. J. Surg..

[38] Magge R., Diamond E.L. (2018). Neurologic complications of gastrointestinal cancer. Cancer Neurology in Clinical Canc. Neurol. Clin. Pract..

[39] SuJata J., Rana S., Sabina K., Hassan M.J., JaiRaJpuRi Z.S. (2013). Incidental gall bladder carcinoma in laparoscopic cholecystectomy: a report of 6 cases and a Review of the literature. J. Clin. Diagn. Res..

[40] Hasan A., Abozied H., Youssef A., Fayad S., Ismail A. (2020). A rare case of collecting duct carcinoma with first presentation of respiratory symptoms. Urology Case Reports.

[41] Mittal R., Jesudason M.R., Nayak S. (2010). Selective histopathology in cholecystectomy for gallstone disease. Indian J. Gastroenterol..

[42] Aloia T.A., Járufe N., Javle M., Maithel S.K., Roa J.C., Adsay V., Coimbra F.J., Jarnagin W.R. (2015). Gallbladder cancer: expert consensus statement. HPB.

[43] Kannan N.S., Kannan U., Babu C.G. (2014). Congenital bilobed gallbladder with Phrygian cap presenting as calculus cholecystitis. J. Clin. Diagn. Res.: J. Clin. Diagn. Res..

[44] Lev-Toaff A.S., Friedman A.C., Rindsberg S.N., Caroline D.F., Maurer A.H., Radecki P.D. (1987). Multiseptate gallbladder: incidental diagnosis on sonography. AJR Am. J. Roentgenol..

[45] Erdogmus B., Yazici B., Ozdere B.A., Akcan Y. (2004). Clinical and ultrasonographical findings in patients with multiseptate gallbladder. Tohoku J. Exp. Med..

[46] Bertozzi M., Bizzarri I., Angotti R., Fusi G., Ceppi S., Di Cara G., Esposito S., Messina M., Molinaro F. (2019). Multiseptate gallbladder in a child. Journal of Pediatric Surgery Case Reports.

[47] Choudhury M., Pujani M., Katiyar Y., Jyotsna P.L., Rautela A. (2014). Idiopathic eosinophilic cholecystitis with cholelithiasis: a report of two cases. Turk Patoloji Derg.

[48] Hasan A, Nafie K, El-Sayed S, Nasr M, Abdulmohaymen A, Baheeg M, Abbadi O (2020). Enterobius vermicularis in appendectomy specimens; Clinicopathological assessment: Cross sectional study. Annals of Medicine and Surgery.

